# Advancements in Bacteriophages for the Fire Blight Pathogen *Erwinia amylovora*

**DOI:** 10.3390/v16101619

**Published:** 2024-10-16

**Authors:** Dufang Ke, Jinyan Luo, Pengfei Liu, Linfei Shou, Munazza Ijaz, Temoor Ahmed, Muhammad Shafiq Shahid, Qianli An, Ivan Mustać, Gabrijel Ondrasek, Yanli Wang, Bin Li, Binggan Lou

**Affiliations:** 1 State Key Laboratory of Rice Biology and Breeding, Ministry of Agriculture Key Laboratory of Molecular Biology of Crop Pathogens and Insects, Zhejiang Key Laboratory of Biology and Ecological Regulation of Crop Pathogens and Insects, Institute of Biotechnology, Zhejiang University, Hangzhou 310058, China; 22316251@zju.edu.cn (D.K.); liupf@zju.edu.cn (P.L.); drmunazzaijaz@zju.edu.cn (M.I.); temoorahmed@zju.edu.cn (T.A.); an@zju.edu.cn (Q.A.); 2 Department of Plant Quarantine, Shanghai Extension and Service Center of Agriculture Technology, Shanghai 201103, China; toyanzi@126.com; 3 Station for the Plant Protection & Quarantine and Control of Agrochemicals of Zhejiang Province, Hangzhou 310004, China; lfshou@163.com; 4 Department of Life Sciences, Western Caspian University, Baku AZ1001, Azerbaijan; 5 Department of Plant Sciences, College of Agricultural and Marine Sciences, Sultan Qaboos University, Al-Khoud, Muscat 123, Oman; mshahid@squ.edu.om; 6 Faculty of Agriculture, University of Zagreb, Svetošimunska Cesta 25, 10000 Zagreb, Croatia; imustac@agr.hr (I.M.); gondrasek@agr.hr (G.O.); 7 State Key Laboratory for Managing Biotic and Chemical Threats to the Quality and Safety of Agro-Products, Institute of Plant Protection and Microbiology, Zhejiang Academy of Agricultural Sciences, Hangzhou 310021, China

**Keywords:** biocontrol, fire blight, genome, phage, plant disease

## Abstract

*Erwinia amylovora*, the causative agent of fire blight, causes significant economic losses for farmers worldwide by inflicting severe damage to the production and quality of plants in the Rosaceae family. Historically, fire blight control has primarily relied on the application of copper compounds and antibiotics, such as streptomycin. However, the emergence of antibiotic-resistant strains and growing environmental concerns have highlighted the need for alternative control methods. Recently, there has been a growing interest in adopting bacteriophages (phages) as a biological control strategy. Phages have demonstrated efficacy against the bacterial plant pathogen *E. amylovora*, including strains that have developed antibiotic resistance. The advantages of phage therapy includes its minimal impact on microbial community equilibrium, the lack of a detrimental impact on plants and beneficial microorganisms, and its capacity to eradicate drug-resistant bacteria. This review addresses recent advances in the isolation and characterization of *E. amylovora* phages, including their morphology, host range, lysis exertion, genomic characterization, and lysis mechanisms. Furthermore, this review evaluates the environmental tolerance of *E. amylovora* phages. Despite their potential, *E. amylovora* phages face certain challenges in practical applications, including stability issues and the risk of lysogenic conversion. This comprehensive review examines the latest developments in the application of phages for controlling fire blight and highlights the potential of *E. amylovora* phages in plant protection strategies.

## 1. Introduction

*Erwinia amylovora* (Ea) is responsible for the devastating fire blight disease affecting the Rosaceae family, including apple (*Malus* spp.), pear (*Pyrus* spp.), quince (*Cydonia* spp.), raspberries and blackberries (*Rubus* spp.) and numerous other crops within this family. The disease is highly transmissible and challenging to control due to its efficient transmission and survival mechanisms, coupled with the lack of effective management strategies. These factors contribute to substantial annual economic losses for farmers worldwide [1,2]. A similar disease, known as Asian pear fire blight, caused by *Erwinia pyrifoliae* has been identified in South Korea [3]. Pear fire blight was firstly identified in North America in the 1780s and is presently been observed in Europe, Africa, Australia, the Middle East, and Asia [4,5,6,7,8,9,10], establishing a global presence.

*E. amylovora* infection typically occurs during blossom opening, and is naturally conveyed by wind, rain, and insects [11,12]. The pathogen subsequently colonizes the xylem vessels, where its pathogenic mechanism involves tissue necrosis through the production of copious amounts of exopolysaccharides (EPSs). These EPSs obstruct the vasculature, impeding the transport of nutrients and water [2]. Additionally, a type III secretion system is employed by *E. amylovora* to insert effector proteins into the cytoplasm of the plant cell [13], inducing cell necrosis [14]. According to European Union (EU) regulations, *E. amylovora* is classified as a protected zone quarantine pest [15]. Similarly, in China, it is listed among the quarantine pests for imported plants. To date, the most effective strategy for managing fire blight remains the implementation of stringent phytosanitary measures [16]. Another efficient approach involves integrated management, which encompasses a range of preventive measures, rigorous phytosanitary practices, and the implementation of preventative measures when the disease has not yet manifested. Additionally, the development of a robust disease control strategy is crucial when the disease has already occurred.

Fungicides, including antibiotics, copper compounds, and aluminum compounds, are commonly employed to prevent and control fire blight before and after flowering in the field. Currently, streptomycin is the most widely adopted bactericide, demonstrating the highest efficacy in reducing fire blight pathogen populations [17]. However, the emergence of antibiotic-resistant bacteria poses a significant challenge to the sustained control of fire blight. Concerns regarding antibiotic residues in leaves and fruits, environmental pollution, and alterations in soil bacterial communities have restricted their usage [18]. Consequently, some European countries have banned antibiotics for plant disease management, and China officially withdrew agricultural streptomycin from its market in 2018 [19,20]. These concerns and limitations in effectiveness have led to a growing demand for non-hazardous and more sustainable agricultural practices in fire blight control. The field of biological control has witnessed increased research activity, with notable progress in the biological control of fire blight.

Bacteriophages (phages), a type of virus that infects bacteria, are emerging as a promising class of biocontrol agents. Numerous *Erwinia* phages have been characterized, and several commercial phage products have been developed [10,21,22,23,24] that are available globally as solutions against fire blight, such as Omnilytics AgriPhage-Fire™ Blight (Salt Lake City, UT, USA Patent No: 4828999) and Enviroinvest Erwiphage PLUS (Kertváros, Hungary registration number: P07 00 600) [25,26]. Furthermore, a novel phage carrier system (PCS) has emerged, and under optimal conditions, this treatment approach is akin to the use of antibiotic streptomycin [27]. Phage therapy is considered an environmentally friendly option for controlling pear fire blight due to its high specificity, low impact on the microbial community balance, and non-hazardous impact on plants and beneficial microbiota, as well as its capability to kill antibiotic-resistant bacteria [21,28,29].

However, it is also important to be aware of the potential environmental stresses that may arise during the process. This paper provides a comprehensive review of recent advancements in the application of phages to mitigate fire blight disease, along with an overview of the potential challenges and prospects of phage therapy.

## 2. Characterization of *E. amylovora* Phages

### 2.1. Isolation of Phages

The concept of utilizing phage therapy to control fire blight disease was first proposed in 1973, when phages capable of lysing *E. amylovora* strains and the yellow saprophytic bacterium *Pantoea agglomerans* were isolated from the soil [30]. Since then, researchers have intensified their efforts to study these phages, focusing primarily on the characterization and application of novel phages for the control of *E. amylovora*. Phages infecting *E. amylovora* represent four morphological categories: myovirus, podovirus, siphovirus, and filamentous phages. The myovirus and podovirus phages are present in greater numbers than the siphovirus or filamentous phages in several American, European, and Asian countries [5,7,8,9,21,24]. Siphovirus phages have been isolated in South Korea, Hungary, and Ukraine [26,31,32], while the siphovirus PhiEaH1 is one of the components of the commercially available *Erwinia* phage cocktail [26]. Recent studies have also demonstrated the potential of filamentous phages, albeit their representing a minority of identified phages, in controlling *E. amylovora* by modulating host fitness. Akremi et al. [10] isolated and characterized four filamentous phages infecting *E. amylovora* in a Tunisian orchard. Research on *E. amylovora* phages commenced in the 1970s [30] and continues to identify new phage candidates with control potential, indicating promising prospects for developing a diverse range of phage-based materials for fire blight control and prevention.

### 2.2. Morphological Features

To comprehensively understand the diversity and distribution of *E. amylovora* phages, it is crucial to examine their morphological characteristics and geographic distribution patterns. Phages exhibit various forms, with a dominant tadpole-like appearance. The head is icosahedral, and the DNA is packaged inside. The head is composed of capsomers and major and minor capsid proteins. Only myoviruses have the contractile sheath covering the tail. Podo- and siphovirus tails are different. The myovirus tail has a central tube that is surrounded by the contractile sheath. The host cell passes through the central space of the core [33,34,35]. Table 1 presents information on the *E. amylovora* phages, including tail shape, head dimension, and total length, which is in high concordance with that of the class Caudoviricetes. Filamentous phages differ in structure, with their viral DNA present in host cells as double-stranded DNA plasmids. The released viral particles contain a circular, single-stranded DNA (ssDNA) encapsulated within a long clathrin cylinder, measuring approximately 7 nm in diameter and 800–2000 nm in length. Transmission electron microscopy (TEM) observations reveal that Caudoviricetes phages constitute the majority (>96%) of Erwinia phages, underscoring their prevalence in this bacterial system.

Table 1 presents a comprehensive list of the *E. amylovora* phages from previously published articles, including their source, morphology, head size, and tail length. An analysis of this data reveals a correlation between the geographical location of the sampling sites and the distribution of phage morphotypes. In South Korea, for instance, podovirus phages accounted for 35.2% of the isolated phage strains, which is higher than the overall average of 24.0%. Conversely, podovirus phages were isolated from Hungary with a percentage of 13.3%, which is less than the average percentage. Additionally, only myovirus phages were found in Spain, while inoviruses were only identified in Tunisia. This indicates that myoviruses remain the predominant phage in the majority of regions. Conversely, siphovirus phages and filamentous phages are only observed in specific locations, and there are no documented instances of siphovirus or filamentous phages in North America, the region where the disease had its origin. This distribution pattern may indicate that the ecological niches of these phage types in certain regions are already occupied by other phage varieties.

### 2.3. Host Ranges

The efficacy of phages as biocontrol agents is largely dependent on the breadth and specificity of their host range. Therefore, a thorough understanding of a phage’s host range is crucial for its effective application in controlling plant pathogens, which typically exhibit narrow host ranges [15,57]. Phages capable of infecting two pathogens at the same time can mitigate two different diseases at the same time, and some phages have been reported to lyse not only *E. amylovora* but also other closely related bacterial species, such as *E. pyrofoliae*, which is a pathogen of the Asian pear tree (*Pyrus pyrifolia*) [58]. The phages pEp_SNUABM_01 and pEa_SNUABM_55, isolated from South Korea, infected only 2 and 6 of 25 *E. amylovora* strains, respectively, whereas they showed a relatively wide host range against *E. pyrifoliae*, infecting 12 and 6 of the 25 *E. pyrifoliae* strains, respectively [47]. Phage mixtures can expand the overall host range and infect a broader spectrum of hosts. For example, among the four phages isolated by Su et al. [50], the phages pEp_SNUABM_03 and 04 exhibited broad-spectrum infectivity, whereas the phage pEp_SNUABM_11 displayed a narrower range but was highly infectious, and the phage pEp_SNUABM_12 could only infect 2 out of 92 strains of *E. amylovora*. However, a mixture of the four phages was able to infect 91 out of 92 strains of *E. amylovora* and all 24 strains of *E. pyrifoliae* [50]. Furthermore, some phages can also infect the epiphytic bacterium *Pantoea agglomerans*, including the phages Joad, Ea2809 [42], RisingSun [38], Y3 [53], and φEa21-4 [59]. Interestingly, *P. agglomerans* was also used as a vector to stably carry phages for the control of the target pathogen, *E. amylovora,* in the study of Boule et al. [21]. The epiphytic bacterium *P. agglomerans* Eh21-5 was used as a vector, and the phages Ea1337-26 and Ea2345-6 caused an 84% and 96% reduction in infections on isolated pear blossom. Previous research has indicated that phages isolated using a single-host bacterial strain tend to have a narrow host range [60,61], whereas phages isolated from mixed-host bacterial strains exhibit a broader host range [5]. Therefore, a more effective biocontrol of pear fire blight can be achieved by strategically selecting *E. amylovora* phages with specific host ranges that complement each other in a phage cocktail.

#### 2.3.1. The Impact of Bacterial EPSs on Host Preference

The production of extracellular polysaccharides (EPSs) is a crucial factor influencing the host range of *Erwinia* phages. This is particularly evident in podoviruses, which have been observed to require the presence of branched-chain starch for the successful infection and colonization of hosts with a higher EPS production [62,63]. Similarly, myovirus and podovirus phages have been shown to exhibit a preference for either high extracellular polysaccharide-producing (HEP) or low extracellular polysaccharide-producing (LEP) bacterial hosts that do not require the addition of supplemental sugar, or the provision of supplemental sugar when grown on artificial media. Myovirus phages typically form clear plaques on LEP hosts and turbid plaques on HEP hosts. Conversely, the majority of podovirus phages demonstrate an inverse preference. Interestingly, myovirus phages show a preference for hosts that produce levan, an additional EPS synthesized by *E. amylovora*. Levan promotes sucrose metabolism and is considered a potential virulence factor [64,65]. However, some phages do not conform to these trends, necessitating further investigation into their infection strategies [62].

#### 2.3.2. The Impact of Phage Source Influences Host Preference

There is a significant variation in host range of *E. amylovora* phages isolated from disparate geographical locations. Generally, phages originating from the same region demonstrated strikingly similar preferences for pathogens from diverse regions. For example, the *E. amylovora* phages Ea214 and Era103, both isolated in Ontario, demonstrated the highest replication rates on hosts from the same region. These phages showed a preference for *E. amylovora* isolates from eastern North America, which resembled the preferences observed in phages from outside North America. Isolates of *E. amylovora* from the western part of North America tend to exhibit a reduced susceptibility to these phages in comparison to isolates from the eastern regions. There are instances of poor phage susceptibility in bacterial isolates from other locations, such as Poland, France, and Israel, while the inconsistency in phage susceptibility among western North American samples is noteworthy [63].

## 3. Genome Analysis of *E. amylovora* Phages

Recent in-depth studies of phage genomes have revealed significant diversity in terms of genome size, GC content, protein coding, and other genomic features. This section aims to provide a comprehensive summary of the research progress made regarding the genomic features, protein function, and existence of tRNA, as well as genetic engineering modifications and safety assessment of *Erwinia* phages. This information will serve as a valuable reference for potential phage applications.

**Figure 1:**
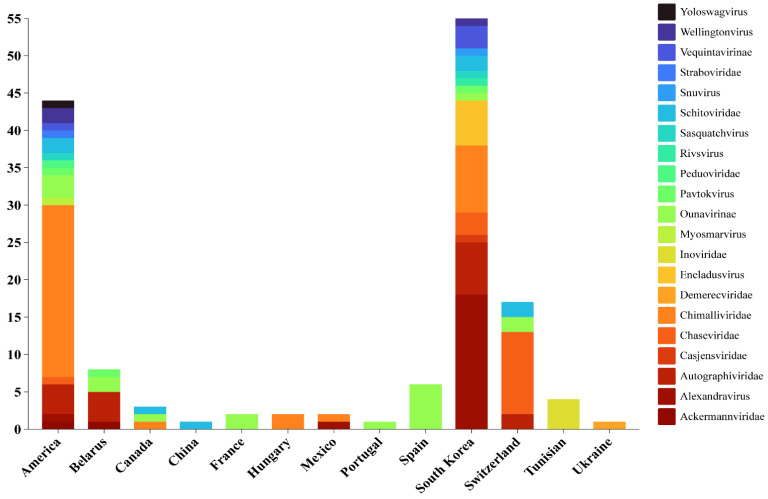
Distribution of phages from various families infecting *E. amylovora* and *E. pyrifoliae* in different countries. The stacked bar chart was constructed utilizing Chiplot (https://www.chiplot.online/; accessed on 22 August 2024).

Based on the registration information from NCBI, Figure 1 provides a holistic perspective on the classification and geographic distribution of *E. amylovora* and *E. pyrifoliae* phages, illustrating the variety of *E. amylovora* phages in different regions and the proportion of each type. The diversity of these phages is manifested globally, particularly in the United States and South Korea. The high number of isolations in America and South Korea may be attributed to their longstanding research history and in-depth comprehension of fire blight disease. This enduring scientific investment could have facilitated the identification of specific phage families, some of which appear to be restricted to these two regions. Moreover, the unique environmental conditions and host diversity in certain areas may also have led to the isolation of distinctive phages, such as the *Demerecviridae* family phages discovered in Ukraine. Further research, especially interdisciplinary studies incorporating environmental and ecological data, will contribute to a more profound understanding of the factors influencing phage distribution and diversity.

Phage Scope was used to analyze the protein functions of 157 *E. amylovora* phage sequences downloaded from NCBI [66], including 9 phages that use the Asian pear fire blight pathogen as a host (Table 2). Most phages have multiple genes in the lysis and replication categories, indicating their ability to effectively lyse host cells and replicate their own genomes, and no lysis-related genes were found in filamentous phages. The number of genes in the assembly and infection categories of phages is also relatively high, emphasizing their ability to form new virus particles and infect host cells. No genes encoding bacterial virulence factors or antimicrobial resistance were detected in the 157 phages analyzed, suggesting their potential suitability for therapeutic applications.

Among the 156 phages, *Ounavirinae* and *Autographiviridae* phages do not encode integrase proteins, suggesting that these two types of phages are strictly lytic, which maximizes their reproduction and spread efficiency by rapidly lysing host cells. Furthermore, *Chimalliviridae* and *Eneladusvirus* phages are all jumbo phages, and the number of defense-related proteins encoded by the giant phages of the *Eneladusvirus* family is significantly higher than that of other phage types. However, the giant phages pEa_SNUABM_48 and pEa_SNUABM_37, which belong to the same family, lack defense-related proteins, revealing the complexity of co-evolution between the host and the phage. Moreover, these related proteins were not also found in the relatively small segment of *Chaseviridae*, *Peduoviridae*, *Inoviridae*, and some *Autographiviridae* phages, suggesting that the genomes of these phages are more streamlined and contain only genes essential for phage replication and assembly.

Table 3 illustrates the genomic features of *Erwinia* phages, which exhibit a considerable range in genome size, from 6608 bp to 358,115 bp, and a similarly broad span in G + C content, spanning from 34.4 to 62.0%, among which jumbo phages with genome sizes of more than 200 kb accounted for 47.6%. This genome size confers a reduced reliance on the host’s replication machinery. The transcription process of jumbo phages is autonomous, facilitated by their own encoded RNA polymerases, independent of the host bacteria [67]. Interestingly, preliminary sequence data indicated the presence of genes encoding an extracellular polysaccharide depolymerase, which is a common feature of *Erwinia* phages but were not detected at the protein level [51,55]; the functions of the majority of phage-predicted ORFs can be broadly categorized into five groups: structural and packaging proteins, nucleotide metabolism-associated proteins, host lysis-related proteins, additional functional proteins, and hypothetical proteins [50].

Some phages also have tRNAs [46]; in particular, mega-phages exhibit a markedly elevated number of tRNAs in comparison to other phages. This phenomenon can be attributed to the fact that the GC content of phages is considerably lower than that of their hosts. Consequently, these phages may leverage their abundant tRNAs to enhance the translation of their genomes [68]. For example, the phages pEa_SNUABM_12, pEa_SNUABM_47, and pEa_SNUABM_50 have been found to encode 32, 35, and 34 tRNAs, respectively [45]. Furthermore, tRNAs are more prevalent in virulent phages than in temperate phages [68]. In contrast, the four filamentous phages are distinguished by a modular organization comprising four modules: replication module, structural module, assembly module, and regulatory module.

The analysis of phage genomes helps us understand the interaction between phages and host bacteria, but can also facilitate the investigation of the synergistic interactions between phages, thereby aiding the identification of suitable candidates. For example, phage Y2 is a myovirus that exhibits a weak virulence but a wide host range due to the absence of the depolymerase gene, whereas phage L1 is a podovirus that possesses the depolymerase gene but has a narrow host range. In the study conducted by Born et al. [69], phage Y2 was genetically modified through homologous recombination. The depolymerase gene *dpoL1* was inserted into the phage Y2 genome, which increased its EPS degradation properties and positive phage infection and killing effect.

**Figure 2:**
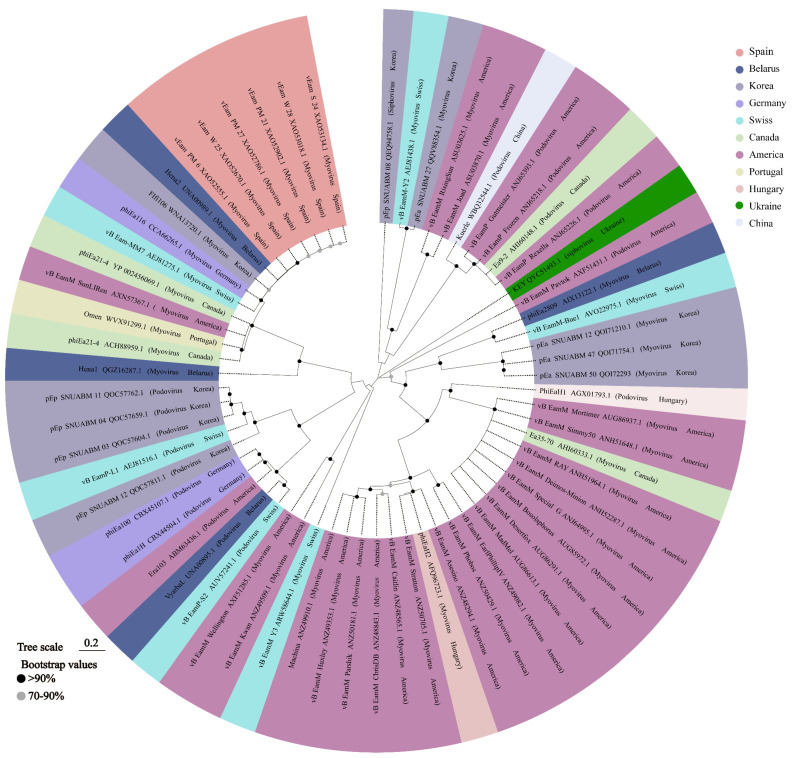
This phylogenetic tree of *E. amylovora* phages was constructed with MEGA 7.0 software by using the maximum composite likelihood method based on the terminase large subunit available in published articles. Nodes show the result of 500 bootstrap replicates.

In order to investigate genetic diversity, sequences available in published articles were used to construct evolutionary trees based on the large subunit of the terminase using a maximum composite likelihood method. The results of this analysis suggest that the phylogenetic relationships among phages within the same family are highly complex. As shown in Figure 2, multiple distinct clades are evident within both the myovirus and podovirus phages, indicating substantial genetic diversity among phages within these taxonomic groups. Interestingly, some phages demonstrate a closer evolutionary affinity despite originating from disparate geographical locations. This observation suggests that phages from different geographical regions, such as those isolated in Switzerland and the United States, may share a higher degree of genetic similarity than previously anticipated. Conversely, phages from disparate geographical regions may display a higher degree of evolutionary divergence. For instance, phages obtained from Spain may possess a distinct evolutionary history compared to those isolated from South Korea.

Generally, *E. amylovora* phages demonstrate a high degree of diversity across all aspects of their genomes. These results not only deepen our understanding of the infection mechanism of *E. amylovora* phages, but can also enhance the utilization of phages in the biological control of pear fire blight. Therefore, future studies should be carried out to elucidate the genetic diversity and functional properties of phages, with the aim of promoting the application of *E. amylovora* phages in a broader range of fields.

## 4. Infection Mechanism of *E. amylovora* Phages

### 4.1. Lytic Activity

The lytic activity of *E. amylovora* phages has been typically illustrated using the one-step growth curves of the phages by measuring the quantity of phages released into the culture medium at various time points [29]. A one-step growth curve typically exhibits a sigmoidal pattern, with an initial lag phase followed by an exponential increase in phage numbers until a plateau is reached, indicating complete lysis of the host cells and the release of progeny phages. The lytic activity of a phage represents a significant aspect of its biological properties, which can be used as a criterion for selecting candidate host bacterial strains. The duration of the latency period directly influences the efficiency of replication, while the magnitude of the burst serves as a pivotal indicator of the phage’s lytic activity. A larger burst size indicates that the phage is more capable of replicating in the host cells [70], thus increasing the probability of infection.

Table 4 presents the published lytic activity data of some *Erwinia* phages. The results indicate that the latency period of all phages except vB_EamM_Deimos-Minion is shorter than 60 min and the range of burst sizes was 20–340 PFU/host cell. The four filamentous phages, as well as the lysogenic phages RH-42-1 and Fifi106, exhibited a high burst size and a short latency period. These characteristics enable phages to replicate and assemble efficiently in host bacterial cells, thereby producing a large number of viral particles in a short period of time. These different lytic patterns imply distinct interaction mechanisms between phages and their host bacteria. Therefore, the efficacy of phage application can be prolonged by selecting an appropriate combination of phages with different lytic patterns and then formulating them into a phage mixture with a high lytic activity for disease control purposes.

A lytic phage normally infects host bacteria by several processes, including the adherence of phages to the host cells, injection of DNA into the host cells, and self-replication in the host cells, causing the death of host bacterial cells. Once a phage has attached to a susceptible host, it will typically commence a lytic or temperate replication strategy. The rigorous lysis of phages has significant potential as a therapeutic strategy due to their prevalence in the environment and self-replicating nature. The killing of host bacteria by phages is primarily through the common holin–lysin system, which results in the lysis of bacterial cells [9]. Endolysin and holin have been documented to inflict damage on the inner cell membrane and the peptidoglycan layer, respectively. In most cases, the function of endolysin needs the assistance of holin, which facilitates the delivery of phage muramidases to the murein sacculus by creating pores in the inner cell membrane and also regulates the precise timing for the lysis of bacterial cells [72,73,74].

### 4.2. Variability in Infection Manner of E. amylovora Phages

Temperate phages have the ability to invade the host bacteria via the lysogenic pathway, whereby the phage genome integrates as a prophage into the host chromosome or the prophage may persist as a plasmid [22,75,76]. There is a heightened consideration of lytic phages as potential candidates for phage therapies. On the other hand, bacteria have evolved a variety of ways and mechanisms to evade phage multiplication at different stages of the infection cycle. One such mechanism is the capability to hinder phage adsorption on the bacterial cell surface [77]. For example, *E. amylovora* produces exopolysaccharides (EPSs), which form a physical barricade on the cell surface, hindering any adherence of the phage and making the bacterium invulnerable to infection [78].

There is a great variability in the infection manner of *E. amylovora* phages from disparate families. For example, podovirus phages infect *E. amylovora* through the degradation of EPSs with a depolymerase enzyme. Some studies have reported that host bacteria lacking EPSs affect the infection rate of podoviruses [39,62]. For instance, the podovirus phage S6 was unable to infect host bacteria lacking cellulose, while lacking cellulose synthesis did not diminish the virulence of the bacterial pathogen [79]. Furthermore, the primary component of the tail structure of phage L1 was identified as the depolymerization enzyme *dpoL1* [52], which has the potential to confer host specificity by interacting with EPS capsules produced by *E. amylovora*. Phage L1 or recombinant *dpoL1* exhibits the ability of EPS degradation, resulting in the cessation of the EPS synthesis of host bacteria [62].

### 4.3. Synergistic Interaction

Phage synergism refers to a phenomenon within a phage cocktail where one phage enhances the properties of another, thereby leading to a higher and/or faster lysis rate of the pathogen. The synergistic outcome of a phage mixture requires one phage to create an environment that enhances the virulence of the second phage [29]. The potential for synergistic interactions between phages is a critical factor to consider when selecting phage strains, especially for the lysis of biofilm-forming bacteria. For example, as demonstrated in the study conducted by Schmerer et al. [80], the phage J8-65 yielded a tailspike colanidase that decomposed the mucoid layer on the bacterial surface, thereby facilitating the entrance of the second T7 phage into the bacterial receptor. The combination of the two phages was found to be 10-fold more effective than T7 alone and approximately 100-fold more effective than J8-65 alone [80]. Furthermore, a synergistic killing outcome was noted following co-infection with the podovirus phage L1 and myovirus phage Y2. Interestingly, phage Y2 could infect all tested hosts with weak virulence, while phage L1 demonstrated a narrow host range with strong virulence. The discrepancy may be mainly due to fact that that phage Y2 lacks a depolymerase that can degrade the host barrier EPSs, whereas phage L1 possesses a depolymerase and also requires the presence of EPSs during host recognition [52].

The application of phage mixtures in place of individual phages represents a general method of extending the host specificity of phage preparations. Despite isolation from the same soil sample in Hungary, only an approximately 6% similarity was found between the siphovirus phages PhiEaH1 and PhiEaH2, revealing potential adaptation to specific host species. Thus, the sequencing of more phage genomes will facilitate the identification of greater diversity, thereby creating opportunities to develop more effective biocontrol agents and phage mixtures against fire blight [26]. In addition, any family of phages that lacks undesirable genes encoding integration-related proteins, host virulence factors, antibiotic resistance determinants, and so forth, could be suitable candidates for a combined approach to fire blight biocontrol [22,31,32,48]. The use of phage therapy can lead to a notable enhancement in the efficacy of therapeutic interventions, particularly through the selection of combinations of phages that exhibit a synergistic effect. Furthermore, a comprehensive analysis of phage genomes can facilitate the discovery of novel phage species that can be employed as biocontrol agents for enhanced disease management. A review of the available studies revealed that the majority of myovirus phages exhibit a broader host range but a reduced virulence compared to podovirus phages. Thus, the combination of such podoviruses with a diverse range of myoviruses represents an appealing approach for the effective biocontrol of fire blight.

## 5. Tolerance to Environmental Stresses

In the practical application of phages, the environmental persistence of bacteriophages is a critical factor in determining the long-term sustainability of their biocontrol effects. Various abiotic factors, including high temperatures, extreme pH levels, and ultraviolet (UV) radiation, can significantly impact the persistence, survival, and stability of phages [81], as well as infection processes such as adherence, penetration, intracellular replication, and amplification within the host bacterial cells [82,83]. Additionally, extreme pH levels can impede the binding of phages to the receptor sites on host bacterial cells by disrupting the function of either the lysozyme enzyme or other structural proteins within the phage capsid [84]. The viability and integrity of phages are significantly modulated by temperature and pH, which can impede key stages of the phage life cycle, including the attachment to host cell receptors, translocation of genetic material, intracellular replication, and progeny virion assembly. At reduced temperatures, the efficiency of genetic transduction into bacterial hosts is diminished. In contrast, elevated temperatures may induce denaturation of the phage capsid proteins, consequently prolonging the latency phase of the phage. These phenomena underscore the pivotal impact of temperature and pH on the therapeutic efficacy of phage-based interventions [84,85,86].

There is a great variety between phages in thermal stability and pH stability. The majority of phages can survive at 25–50 °C and a pH of 4–9 [9,48,49,71] and show good activity under conditions of a high temperature, strong alkali, and prolonged UV radiation. For example, phage EP-IT22 is active in a pH of 4–11 and inactivated at pH 12, while phage ΦFifi106 remains active at a pH of 4–11 [48]. Phage RH-42-1 showed high temperature resistance after a 60 min treatment at 60 °C [9]. EP-IT22 was biologically active in range of −80 °C to 60 °C, whereas incubating it at 70 °C for 1 h killed it [44]. In addition, filamentous phages have better heat tolerance properties, and a considerable loss of phage titer was only noted at 80 and 100 °C after incubating for 1–6 h.

UV irradiation is commonly considered as the most essential factor for the decrease in and loss of phage operation in the natural environment by impacting the longevity of phages in the plant phyllosphere [87,88,89,90]. Indeed, it has been known that UV radiation can directly destroy free viruses through the degradation of their proteins, changes in nucleic acid structure, and reductions in phage infectivity [91]. In particular, the permanent effect induced by shorter wavelengths was observed on the genomic material, resulting in both the alteration of viral proteins and the development of deadly photoproducts [91]. However, the sensitivity of phage particles to UV radiation can be defeated by adopting diverse strategies, including high-titer phages at dawn or at nighttime when radiation is restricted [84].

Indeed, Born et al. [92] revealed that natural extracts of carrot, red pepper, beet-root, and casein and soy peptones in solution, as well as pure substances such as astaxanthin, aromatic amino acids, and Tween 80 can significantly prolong the half-life of UV-irradiated phage particles without negatively affecting phage viability or infectivity. Kristi et al. [93] found that kaolin and 4.5% polysorbate 80 can enhance phage stability and uptake efficacy in plants under UV stress. These results revealed the importance of the protective formulation of *Erwinia* phage-based formulations against environmental stress [94].

## 6. Application

### 6.1. Phage Cocktails

Some phages exhibit a high degree of specificity, infecting only one or a few strains within a single bacterial species. This characteristic is advantageous for sustaining a balanced microbial ecosystem [29]. However, the ongoing evolutionary arms race between phages and bacteria has led to the development of various phage resistance mechanisms in bacteria, such as the inhibition of phage adsorption and DNA entrance, abortive infection, the CRISPR/Cas immune system, and restriction–modification systems [95]. By employing a diverse array of phages, it is less likely that the target bacteria will develop resistance to all phages simultaneously. Moreover, phages typically exhibit host specificity, with different phages being utilized in aiming at various pathogens (different species causing similar diseases or different strains within the same species). Formulating a cocktail of phages with various hosts can broaden the host range [96], while phage cocktails are capable of targeting pathogenic bacteria with great efficiency, having been applied on multiple occasions since the 1990s [97]. Lytic phage mixtures compensate for the limitations of individual phage host ranges and prevent the development of phage-resistant bacteria [29]. Furthermore, there may occur synergistic interactions between phages in the mixture, where one enhances the properties of the other [52,71]. In particular, phage mixtures can improve phage adsorption and the lysis of the pathogen. In contrast to the significant environmental and human health risks of antibiotics and copper-based strategies, phage mixtures display a broad range of host activities by recognizing various receptors, thereby reducing the likelihood of resistance development in host bacteria [29,76]. Therefore, it can be inferred that phage mixtures are promising antimicrobial agents with distinctive features of specificity by targeting drug-resistant pathogens without harming host plants or animals and their symbiotic microbial communities.

#### 6.1.1. Development of Cocktail Formulation Methods

The development of phage cocktails involves several strategic considerations to ensure a broad coverage of target pathogens with diverse infection mechanisms and a complementary infectivity towards the host pathogens. Ideally, phages within a cocktail should have different receptors, high adsorption rates, short latency periods, and large burst sizes. Another factor affecting phage infection is the ability of phages to adsorb to receptors on the surface of bacterial cells. These receptors include outer membrane proteins, teichoic acids, flagellar filaments, capsules or slime layers, and lipopolysaccharides. When formulating a phage cocktail, these phage–host interactions ought to be taken into account. It is generally preferable to use phages with different receptors, as different single-point mutations are unlikely to occur concurrently [29].

Additionally, the use of genetically engineered phages can be applied to formulate synthetic phage cocktails, targeting specific bacterial species within a mixed population. For example, as previously mentioned, phage Y2 has been engineered to improve its killing efficiency and also serves as a luciferase reporter gene through homologous recombination [98]. Various studies have investigated the possibility of expanding or altering the host range of phages by genetically modifying the phage receptor-binding proteins (tail fibers). Multiple genetically modified phages can then be formulated into a mixture capable of lysing specific bacteria. However, it is not feasible completely to prevent the evolution of new phage-resistant plant pathogens by creating phage cocktails, due to the ongoing arms race between phages and bacteria. Therefore, it is necessary to continuously monitor plant pathogens and the corresponding reformulation of phage cocktails to ascertain their effectiveness against newly emerging phage-resistant plant pathogens. Fortunately, the abundance of phages in nature makes it possible to isolate new phages capable of killing these emerging phage-resistant plant pathogens.

#### 6.1.2. Formulation and Commercial Preparation

Phage cocktail therapy represents a multifaceted approach to biocontrol, with environmental and host bio-compatibility as well as an increased lysis efficiency against drug-resistant bacteria through the combination of multiple phages. The results of the existing studies indicate that the effect of phage mixtures is superior to that of individual strains in the management of plant diseases caused by varieties such as *Xylella fastidiosa* [99,100] and *Ralstonia solanacearum* [101]. Furthermore, some phages exhibit a synergistic effect in the biocontrol of fire blight. For example, the commercial Erwin phage and its successor Erwin PLUS have been developed and successfully applied in production [102], thereby demonstrating the practical effectiveness of phage mixtures. The product is comprised of two components, a phage and a UV-protective solution, while the formulation of Erwiphage includes two siphoviruses, PhiEaH2 and PhiEaH1 [25,26]. Therefore, phage cocktail therapy is anticipated to become a crucial complement to traditional antibiotic therapy in controlling fire blight disease.

### 6.2. Phage–Carrier System

Although a small number of *E. amylovora* phages have a narrower host spectrum, the majority of them can infect *P. agglomerans* [8,21,23,59], which fills the same ecological niche on the stigma without pathogenicity and contends with *E. amylovora* during the blossom colonization period. The benefit of employing bacteria as a phage vector is that the phages reproduce within the infected carrier bacteria. Since many *Pantoea* spp. isolates produce carotenoids [103], the reproduced phages can be protected from harmful ultraviolet radiation and other potential environmental factors. After the carrier is lysed, the phages are discharged onto the colonized blossoms, which may lead to direct delivery to the co-located target *E. amylovora*. Thus, *P. agglomerans* is a promising biocontrol agent and a vector for phages. This concept is based on the assumption that sensitive strains of *P. agglomerans* can be used as vector organisms for the multiplication and delivery of phages to aimed-at bacteria.

Lehman firstly reported a broad host range of *E. amylovora* phages in combination with the phage vector *P. agglomerans* [27]. The phage–carrier system (PCS) represents a novel BCA that employs a two-pronged therapeutic approach for the mitigation of fire blight [59]. This approach involves the use of phages that have the capability of infecting both the antagonistic bacteria, namely *P. agglomerans*, which serves as the phage vector, and *E. amylovora*. A study combining a PCS with *P. agglomerans* and *E. amylovora* phages demonstrated that, under suitable conditions, this treatment is comparable to the application of streptomycin [1]. Comparing the efficiency of different phage + carrier (*P. agglomerans* EH21-5) combinations with other commercial biocontrol products (BlightBan^®^ A506, BlightBan^®^ C9-1, Nufarm, Melbourne, Australia), two phage–carrier combinations (ΦEa21-4 + EH 21-5 and ΦEa46-1 + EH 21-5) reduced the incidence of fire blight disease in pears by 50% and 63%, respectively, similar to the streptomycin control [24]. However, it is worth mentioning that similar results were also obtained using BlightBan^®^ C9-1 alone [27]. Although the phage–vector combination must be in a stable formulation to maintain its viability and effect, PCSs offer a potential avenue for reducing the emergence of phage resistance [27].

**Figure 3:**
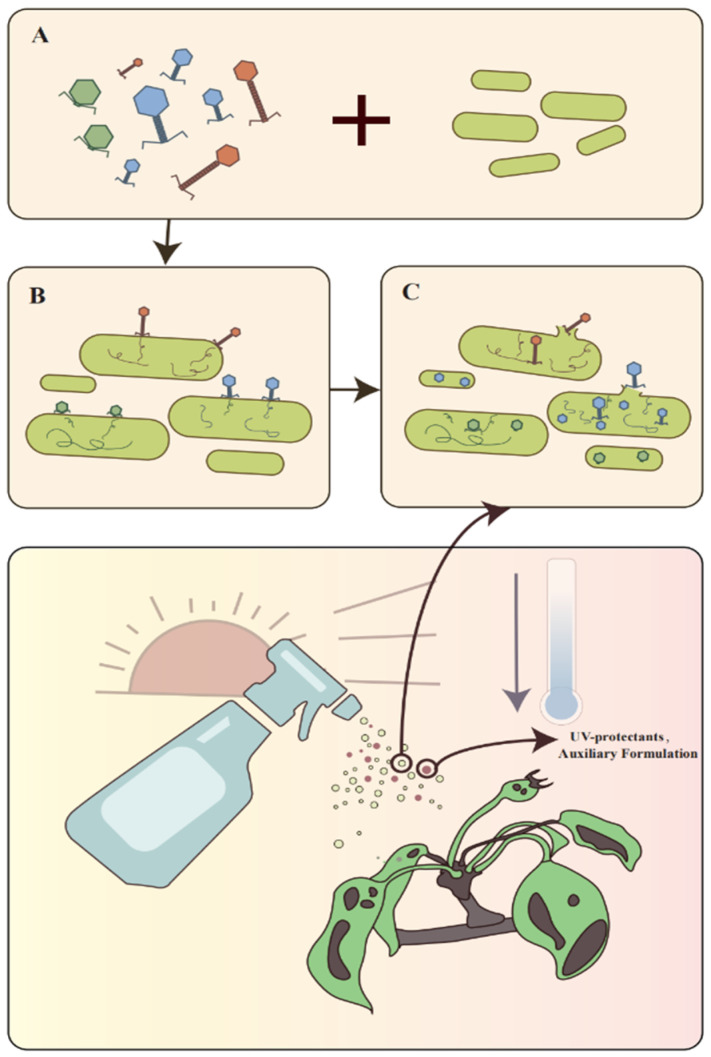
The figure illustrates an integrated control strategy based on a phage–carrier system and phage cocktail approach. (**A**) Different phages binding to vector bacteria; (**B**) phage infestation of vectors; and (**C**) phage release. The strategy employs a combination of three key elements: phages, UV protectants, and an auxiliary formulation.

Therefore, it is proposed that a combination of phage cocktail therapy and PCSs, as illustrated in Figure 3, be employed to create a phage collection comprising well-matched phages from different families, which have been subjected to rigorous evaluation. It is also recommended that may tests should be carried out to elucidate the host range of the various phages and their correlation with the bacterial phylogeny in order to produce rational phage applications based on strain distribution. Examples of such combinations include those with complementary properties or synergistic effects. In formulating phage mixtures, it is preferable that the mixtures consist of phages that recognize different receptors and can infect *P. agglomerans*. The selected phage strains should then be combined with *P. agglomerans* and properly treated; for example, drying and freeze-drying [104] should be employed, followed by a combination of UV-protective materials as well as protective agents [94] to control fire blight.

### 6.3. Prospects of Filamentous Phage Applications

Given the diversity and interactions of the constituent phages in the cocktail, it is essential to devote considerable attention to studying their potential mechanisms of infection. Furthermore, it is widely held that temperate phages should not be considered for applications since they tend to specialize or generalize in the transduction of pathogenic determinants [29]. However, some studies have put forward the suggestion that filamentous phages may have certain advantages over lysogenic phages. To date, phage-based outdoor spray treatments may be limited by stability issues, such as those caused by sunlight or drying, which are compensated for by the high stability of temperate phages [4]. Moreover, the transducing temperate phage A25 can acquire lytic characteristics through an escape from lysogeny, harnessing the potential of such traits to maintain phages within a lytic life cycle and ensuring they always kill their bacterial hosts upon infection. In addition to utilizing their own characteristics to influence host survival [105], Sharma et al. [106] have also suggested that filamentous phages have the potential to act as biotechnological instruments for introducing genes that encode for restriction enzymes or virulence factors, including *csrA* and *ompF*, into bacterial hosts. Furthermore, they can be engineered to combine specific antigens or silver nanoparcticles [107], thereby offering a strategy to counteract pathogenic bacteria. Consequently, filamentous phages hold significant potential in the field of agricultural plant disease control.

### 6.4. Factors Affecting the Efficacy of Phage Applications

There is the significant issue of the long-term storage and transience of phages. In the rhizosphere, different soil matrixes and soil pH values and the low diffusivity of moisture may restrict the employment of phage mixtures as biocontrol agents. Compared to the rhizosphere, the phyllosphere is conducive to greater destructive effects on phages due to sunlight and temperature. The choice of stabilizers is crucial for phage formulations, as phages, composed mainly of genetic material encapsulated by their protein coats, share similarities with protein-based drugs in terms of formulation. Currently, substances considered as stabilizers include sugars (such as sucrose) and polymers (such as polyethylene glycol) [29]. However, as a result of the varying sensitivity of different phages to chemical and physical conditions, no universal stabilization strategy has been found to apply broadly. This is particularly challenging when contemplating the long-term storage of mixtures composed of phages with diverse stabilities. Therefore, the key task is to ensure that each phage in the mixture maintains its required efficacy within the predetermined storage period.

Optimizing the efficacy of phage applications requires a multifaceted approach to address the challenges of long-term storage, the transience of phages under different environmental conditions, and the variability in phage performance across plant cultivars. The exploration of strategies such as the adoption of non-toxic bacterial carriers, protective formulations, and optimal application timing represents a proactive step towards enhancing phage stability and activity. Despite the individual sensitivity of phages to chemical and physical conditions, the ongoing research and development of universal stabilization strategies is imperative to ensure the maintenance of phage potency throughout the intended storage period. Continuous innovation in this field is essential to harness the full potential of phage therapy in agriculture, ultimately contributing to more sustainable and effective plant disease management practices.

## 7. Challenges and Perspectives

Phage therapy has been noted as an excellent measure in controlling *E. amylovora* infections in Rosaceae plants. However, on the other hand, the specific application of *E. amylovora* phages for the control of fire blight is still beset with a great variety of challenges and limitations. A scientific basis for the rational use of phages in biocontrol can be established by understanding these challenges and limitations, and this will also indicate the direction of future research.

### 7.1. Risks of Genetic Engineering

The application of genetic engineering serves to further enhance the potential of beneficial microorganisms in the field of effective disease management. Indeed, genetic engineering has been employed to modify phages, enhancing their resistance to environmental stresses and broadening their host range. This is exemplified by the study of Born, in which the insertion of the *dpoL1* gene encoding a depolymerization enzyme into the genome of phage Y2 increased its EPS degradation properties and showed positive effects on phage infection and killing [98]. However, genetic engineering also presents biosafety challenges. The potential ecological impacts and interference with natural ecosystems of genetically modified phages released into the environment are difficult to predict and control with current technologies. More and more studies have been carried out to assess the risks and benefits of genetically engineered phages. By doing this, the potential of phages in biocontrol can be better utilized while their potential risks are reduced, thereby providing new solutions for safe, precise, and sustainable agriculture and disease management.

### 7.2. Risks for Plant, Soil, and Microbiome

Polyvalent phage mixtures may pose potential risks to non-target bacterial populations within the plant microbiome, although such risks are likely to be relatively minor. While these phages can target a variety of plant pathogens, they also have the potential to inadvertently kill endophytic bacteria [29]. *E. amylovora* phages may indirectly affect the plant microbiome by altering interbacterial competition or directly shape the composition of the microbiome by influencing the evolutionary processes and population sizes of bacterial communities. In addition, fire blight primarily occurs on leaves and is a foliar disease, while most soil-isolated phages are derived from the soil. Rhizosphere *E. amylovora* phages may regulate the structure of soil bacterial communities and the cycling of organic matter [108]. Phages could potentially impact soil nutrient networks by driving mutations or modulating the gene expression of selected bacterial phyla, thereby affecting the availability of plant nutrients.

### 7.3. Phage–Plant Interactions

The traditional view is that phages do not interact directly with plants. However, the recent identification of phage-like genes within plant genomes suggests that they may have been acquired from bacteria through horizontal gene transfer [57]. The implications of this horizontal transfer are yet to be fully understood, and it remains uncertain whether phages can enter the plant body through natural orifices or wounds. If they do, the duration of their activity within the plant and whether they maintain their original virulence are also unknowns. Furthermore, Nagy et al. [109] reported the translocation of *E. amylovora* phages from roots to the aboveground tissues of apple seedlings, which was detected using real-time quantitative PCR technology. Similarly, when phages were applied to the leaves and stems of plants, their presence was also detected in the roots. Different types of phages exhibit significant differences in their mechanisms of action, and their behavior in vivo and in vitro may vary. Therefore, further research is required to better comprehend the activity of phages within plants, particularly their interactions with both bacteria and plants. In this tripartite interaction, each participant may influence the final outcome of the interaction dynamics.

### 7.4. Challenges in Field Application

The majority of the current research is conducted under laboratory or greenhouse conditions, which are significantly different from variable field conditions, making it difficult to directly translate the research findings into practical applications. Moreover, true field trials are relatively scarce. Additionally, various researchers have used diverse methods to simulate the effects of phages, but there is a lack of a unified assessment system, making the results impossible to compare. Phages can be applied through various methods such as seed coating, soil suspension, or plant spray [110], but each method must overcome the challenges of environmental factors, such as ultraviolet radiation, desiccation, and chemical and biological factors in the soil, all of which may reduce the efficacy of the phages [111]. Generally, phages should be used during the flowering period to prevent infection rather than curing infected trees where bacteria have already spread to the xylem. Due to the non-motility of phages, they rely on passive diffusion to contact the host bacteria, which requires spraying to achieve a uniform and dense coverage to increase the chances of encountering the pathogen. If the coverage is uneven or insufficient, the phages may not effectively infect the invading pathogens, thereby affecting the efficacy of the treatment. Therefore, ensuring the stability and effectiveness of phages under field conditions requires further research and innovative strategies.

### 7.5. Perspectives on Future Directions

To date, a multitude of research has demonstrated that a spectrum of substances can efficaciously shield phages from ultraviolet radiation. Furthermore, the regular application of drip irrigation or watering may serve as a mechanism to sustain elevated phage concentrations in the rhizosphere. Optimization can be pursued not only in the modality of application but also in the evolutionary enhancement of phages. Beyond the artificial modification of phages to broaden their host range and augment their lytic efficiency, the artificial evolution of phages to bolster their resilience against ultraviolet damage is a promising avenue of exploration. A recent study by Tom et al. has delved into the evolutionary adaptation of phages to withstand ultraviolet radiation [112].

In recent years, the identification of numerous novel phages with significant biocontrol potential has heightened the need for a standardized efficiency-testing protocol. Presently, the methodologies for evaluating the efficacy of phage therapy are diverse, yet there exists no universally accepted standard. Some research is predicated on the visual appraisal of symptoms, while other studies concentrate on quantifying plant biomass. A minority of studies have endeavored to quantify both phages and bacteria [21]. The current state-of-the-art technique for precision involves the quantification of phage DNA via quantitative PCR, normalized against the bacterial count measured in colony-forming units [102]. This approach not only affords a more profound comprehension of the interplay between phages, bacteria, and plants, but also presents a more holistic viewpoint in contrast to methods that solely depend on visual diagnostics or the enumeration of bacteria and phages. In order to transcend the constraints of the current methodologies, it is imperative to further investigate and refine the application of high-precision technologies like qPCR to augment the fidelity and expedience of the assessment process.

## 8. Conclusions

The evidence presented in this study suggests that *E. amylovora* phages have great potential to be a valuable resource for the control of fire blight, which can be attributed to the absence of antibiotic resistance genes and genes related to virulence factors, as well as a wide host range. Furthermore, numerous studies have demonstrated that the cocktail therapy of using multiple *E. amylovora* phages can effectively mitigate the limitations associated with single-phage treatments, such as a narrow host range and environmental susceptibility. In addition, the successful use of phage–carrier systems, which combine the advantages of antagonistic bacteria and phages to increase the environmental stability and delivery efficiency of phages through non-pathogenic host bacteria such as *P. agglomerans*, represents a promising avenue for future research. To fully realize the potential of phages as effective, safe, and sustainable biocontrol agents, it is essential to continue improving their stability and host range, assess their behavior in natural environments, and develop new phage combinations and formulation technologies.

## Figures and Tables

**Table 1:** Morphological features of *E. amylovora* phages mentioned in the article.

Phage Name	Region	Morphology	Head Size (nm)	Tail Size (nm)	References
phiEa104	USA	Myovirus	71.56 ± 2.20	114.42 ± 2.51	[36]
phiEa116	USA	Myovirus	73.36 ± 1.89	114.62 ± 2.28	[36]
vB_EamM_RAY	USA	Myovirus	128 ± 5.96	159 ± 11	[24]
vB_EamM_Simmy50	USA	Myovirus	-	-	[24]
vB_EamM_Special G	USA	Myovirus	-	-	[24]
vB_EamM_Deimos-Minion	USA	Myovirus	-	-	[24]
vB_EamM_Bosolaphorus	USA	Myovirus	-	-	[24]
vB_EamM_Desertfox	USA	Myovirus	-	-	[37]
vB_EamM_MadMel	USA	Myovirus	-	-	[37]
vB_EamM_Asesino	USA	Myovirus	-	-	[37]
vB_EamM_Alexandra	USA	Myovirus	-	-	[37]
vB_EamM_Mortimer	USA	Myovirus	-	-	[37]
vB_EamM_SunLIRen	USA	Myovirus	-	-	[37]
vB_EamM_Wellington	USA	Myovirus	-	-	[37]
vB_EamM_RisingSun	USA	Myovirus	-	-	[38]
vB_EamM_Joad	USA	Myovirus	-	-	[38]
vB_EamM_Caitlin	USA	Myovirus	-	-	[38]
vB_EamM_ChrisDB	USA	Myovirus	-	-	[38]
vB_EamM_EarlPhillipIV	USA	Myovirus	-	-	[38]
vB_EamM_Huxley	USA	Myovirus	-	-	[38]
vB_EamM_Kwan	USA	Myovirus	-	-	[38]
vB_EamM_Machina	USA	Myovirus	-	-	[38]
vB_EamM_Parshik	USA	Myovirus	-	-	[38]
vB_EamM_Phobos	USA	Myovirus	-	-	[38]
vB_EamM_Stratton	USA	Myovirus	-	-	[38]
vB_EamM_Yoloswag	USA	Myovirus	-	-	[38]
phiEa100	USA	Podovirus	61.42 ± 2.14	-	[39]
Era103	USA	Podovirus	-	-	[39]
vB_EamP_Pavtok	USA	Podovirus	-	-	[38]
vB_EamP_Frozen	USA	Podovirus	-	-	[38]
vB_EamP_Gutmeister	USA	Podovirus	-	-	[38]
vB_EamP_Rexella	USA	Podovirus	-	-	[38]
Micant	Belarus	Podovirus	56.39 ± 2.69	-	[40]
Loshitsa2	Belarus	Podovirus	59.80 ± 2.60	-	[40]
VyarbaL	Belarus	Podovirus	-	-	[41]
phiEa2809	Belarus	Myovirus			[42]
Hena1	Belarus	Myovirus	72.36 ± 5.38	126.28 ± 5.27	[7]
Hena2	Belarus	Myovirus	-	-	[41]
phiEa21-4	Canada	Myovirus	-	-	[5]
vB_EamM_Ea35-70	Canada	Myovirus			[5]
Kuerle	China	Podovirus			[43]
Ea1594-24	Colombia	Myovirus	48	74	[21]
Ea21-4g	Colombia	Myovirus	50	74	[21]
Ea2345-6	Colombia	Myovirus	54	77	[21]
Ea1615-26	Colombia	Myovirus	62	105	[21]
Ea2345-19	Colombia	Myovirus	65	113	[21]
Ea1594-26	Colombia	Myovirus	72	97	[21]
Ea1598-6	Colombia	Myovirus	80	136	[21]
Ea1337-26	Colombia	Podovirus	53	14	[21]
Ea1598-19	Colombia	Podovirus	60	20	[21]
phiEa1H	Germany	Podovirus	59.89 ± 1.49	-	[36]
ΦEaH2B	Hungary	Myovirus	57 ± 7	60 ± 39	[23]
ΦEaH2A	Hungary	Myovirus	69 ± 7	107 ± 11	[23]
ΦEaH1A	Hungary	Myovirus	70 ± 3	117 ± 4	[23]
ΦEaH4B	Hungary	Myovirus	70 ± 9	98 ± 18	[23]
ΦEaH7A	Hungary	Myovirus	71 ± 8	99 ± 7	[23]
ΦEaH12B	Hungary	Myovirus	72 ± 4	103 ± 4	[23]
ΦEaH5K	Hungary	Myovirus	73 ± 4	107 ± 9	[23]
ΦEaH5B	Hungary	Myovirus	74 ± 5	104 ± 9	[23]
ΦEaH7B	Hungary	Myovirus	77 ± 5	108 ± 6	[23]
ΦEaH4A	Hungary	Myovirus	78 ± 5	108 ± 10	[23]
ΦEaH5K	Hungary	Myovirus	-	-	[23]
ΦEaH11	Hungary	Podovirus	55 ± 2	13 ± 2	[23]
ΦEaH9B	Hungary	Podovirus	61 ± 7	9 ± 3	[23]
PhiEaH1	Hungary	Siphovirus	-	-	[26]
PhiEaH2	Hungary	Siphovirus	-	-	[25]
EP-IT22	Italy	Myovirus	90 ± 5	100 ± 10	[44]
pEa_SNUABM_47	South Korea	Myovirus	127 ± 6	127 ±3	[45]
pEa_SNUABM_12	South Korea	Myovirus	130 ± 5.9	126.7 ± 2.6	[45]
pEa_SNUABM_32	South Korea	Myovirus	130 ± 6	169 ±7	[46]
pEa_SNUABM_31	South Korea	Myovirus	139 ± 5	196 ± 11	[46]
pEa_SNUABM_48	South Korea	Myovirus	140 ± 2	150 ± 17	[46]
pEa_SNUABM_27	South Korea	Myovirus	69 ± 3	115 ± 2	[46]
pEp_SNUABM_01	South Korea	Myovirus	78.29 ± 0.91	-	[47]
Fifi106	South Korea	Myovirus	79.8 ± 4.3	114.1 ± 5.2	[48]
pEa_SNUABM_55	South Korea	Myovirus	81.88 ± 2.20	6	[47]
pEa_SNUABM_50	South Korea	Myovirus	-	-	[45]
Ea46-1-A1	South Korea	Podovirus	-	-	[48]
phiEaP-8	South Korea	Podovirus	75	-	[49]
pEp_SNUABM_04	South Korea	Podovirus	55 ± 3	16 ± 2	[50]
pEp_SNUABM_03	South Korea	Podovirus	56 ± 2	17 ± 2	[50]
pEp_SNUABM_11	South Korea	Podovirus	56 ± 3	18 ± 1	[50]
pEp_SNUABM_12	South Korea	Podovirus	63 ± 2	17 ± 1	[50]
pEp_SNUABM_08	South Korea	Siphovirus	62 ± 4	190 ± 12	[31]
Omen	Portugal	Myovirus	2 ± 5	112 ± 9	[51]
vEam_PM_21	Spain	Myovirus	59.82 ± 3.98	94.56 ± 7.45	[15]
vEam_PM_6	Spain	Myovirus	61.11 ± 5.06	93.02 ± 3.31	[15]
vEam_PM_27	Spain	Myovirus	63.46 ± 4.62	101.92 ± 4.62	[15]
vEam_W_25	Spain	Myovirus	63.85 ± 5.00	94.23 ± 9.23	[15]
vEam_S_24	Spain	Myovirus	69.28 ± 5.32	94.22 ± 4.53	[15]
vEam_W_28	Spain	Myovirus	78.63 ± 7.41	102.77 ± 5.4	[15]
vB_EamP_Y2	Switzerland	Myovirus	67	124	[52]
vB_EamP_M7	Switzerland	Myovirus	77	116	[52]
vB_EamM_Y3	Switzerland	Myovirus	129 ± 4	192 ± 12	[53]
vB_EamM-Bue1	Switzerland	Myovirus	79 ± 2	126 ± 7	[54]
vB_EamP_L1	Switzerland	Podovirus	58	-	[52]
vB_EamP_S6	Switzerland	Podovirus	66	-	[52]
vB_EamP-S2	Switzerland	Podovirus	64 ± 5	-	[55]
PEar1	Tunisia	Inovirus	-	-	[10]
PEar2	Tunisia	Inovirus	-	-	[10]
PEar4	Tunisia	Inovirus	-	-	[10]
PEar6	Tunisia	Inovirus	-	-	[10]
TT10-27	Ukraine	Podovirus	71.3	22	[56]
KEY	Ukraine	Siphovirus	80 ± 1	169 ± 10	[56]

“-”: data unavailable.

**Table 2:** Classification and protein functional analysis of *E. amylovora* phages.

Phage	Unknown	Integration	Immune	Regulation	Lysis	Packaging	Replication	Assembly	Infection	Hypothetical
Ackermannviridae										
vB_EamM-Bue1	95	2	1	6	9	9	21	18	14	49
phiEa2809	93	2	1	6	9	7	21	18	14	50
Alexandravirus										
pEa_SNUABM_1	254	2	3	3	4	10	15	8	23	11
pEa_SNUABM_16	262	2	3	3	4	9	15	8	23	10
vB_EamM_Alexandra	255	3	3	2	7	9	17	6	25	12
pEa_SNUABM_3	253	3	3	2	3	10	16	9	24	11
pEa_SNUABM_32	252	2	4	3	5	10	15	8	22	10
pEa_SNUABM_2	253	2	2	2	4	9	16	9	24	11
pEa_SNUABM_22	260	3	3	3	4	9	15	8	23	11
pEa_SNUABM_17	242	3	3	3	4	10	16	8	24	10
pEa_SNUABM_33	251	2	3	4	4	11	16	10	24	10
pEa_SNUABM_35	253	2	3	3	4	9	16	8	24	11
pEa_SNUABM_30	244	2	3	4	4	11	17	9	24	12
pEa_SNUABM_28	264	2	3	3	4	9	15	8	23	11
pEa_SNUABM_40	253	3	3	2	3	11	17	9	24	10
pEa_SNUABM_18	260	2	3	3	4	9	15	8	23	10
pEa_SNUABM_20	252	3	3	2	3	10	16	9	24	10
pEa_SNUABM_23	253	3	3	2	3	10	16	9	24	10
pEa_SNUABM_31	253	3	3	2	3	10	16	9	24	10
pEa_SNUABM_39	253	2	2	2	4	9	16	9	24	11
vB_Ea_2910A	247	2	3	4	4	13	19	9	24	10
pEa_SNUABM_36	253	2	3	3	4	9	16	8	24	11
Autographiviridae										
phiEa100	11	0	3	1	5	7	7	5	5	9
phiEa1H	11	0	3	1	5	7	7	5	5	9
vB_EamP-S2	13	0	4	0	4	5	5	5	5	9
Era103	9	0	3	2	5	7	7	5	5	9
Tapenade	12	0	4	0	4	5	5	5	5	9
VyarbaL	12	0	4	0	4	5	5	5	5	9
pEp_SNUABM_12	17	0	1	0	3	4	8	7	6	4
pEp_SNUABM_03	21	0	0	3	3	4	6	7	6	3
pEp_SNUABM_10	21	0	0	3	3	4	6	7	6	3
pEp_SNUABM_09	20	0	0	3	3	4	6	7	6	3
pEp_SNUABM_04	19	0	0	3	3	4	6	7	6	3
pEp_SNUABM_11	18	0	0	3	3	4	6	7	6	3
Stepyanka	13	0	0	4	4	4	7	8	6	4
vB_EamP-L1	10	0	0	4	3	5	7	8	6	4
pEa_SNUABM_57	11	0	0	2	3	4	7	7	7	5
Loshitsa2	21	0	1	0	5	5	5	5	6	4
Micant	21	0	1	0	5	5	5	5	6	4
Casjensviridae										
pEp_SNUABM_08	36	0	2	1	3	3	3	8	10	13
Chaseviridae										
vB_EamM-Y2	26	1	0	1	3	4	8	6	13	18
Papaline	29	1	0	1	3	4	8	7	14	16
Calisson	31	2	0	0	3	4	7	6	14	16
Fougasse	29	1	0	0	3	3	7	6	14	16
Nougat	29	1	0	0	3	3	7	6	14	16
Mauresque	28	1	0	1	3	2	7	6	14	16
Faunus	29	1	0	1	4	3	7	6	14	14
Aioli	29	1	0	0	3	4	7	6	14	14
Navette	27	1	0	0	3	2	7	6	14	16
Farigoule	27	1	0	0	3	2	7	6	14	16
Orgeat	29	1	0	0	3	2	7	6	14	16
Fifi440	40	1	0	0	3	3	7	5	4	15
Berlingot	29	1	0	0	3	2	7	6	14	16
Fifi451	30	1	0	0	3	3	7	5	14	15
pEa_SNUABM_27	28	1	0	0	3	2	7	5	14	14
Chimalliviridae										
Derbicus	107	3	3	0	7	4	19	35	32	24
vB_EamM_Earl	107	3	3	0	7	4	19	35	32	24
vB_EamM_Phobos	107	2	4	0	8	4	20	39	36	22
vB_EamM_MadMel	180	3	1	4	8	6	26	25	30	37
vB_EamM_Mortimer	187	3	1	4	8	4	27	25	29	35
Rebecca		3	1	3	8	4	25	26	31	36
vB_EamM_Deimos-Minion	183	3	1	3	8	5	25	25	30	35
vB_EamM_SpecialG	182	3	1	4	8	5	27	24	28	37
vB_EamM_Desertfox	180	3	1	4	8	4	26	25	30	36
vB_EamM_Bosolaphorus	183	3	1	3	8	4-	26	25	30	35
vB_EamM_RAY	181	3	1	3	8	4	25	24	28	36
vB_EamM_Simmy50	181	3	1	4	8	4	26	24	28	36
Ea35-70	185	3	1	3	8	4	25	24	27	35
vB_EamM_Earl	107	3	3	0	7	4	19	35	32	24
pEa_SNUABM_38	106	3	2	0	7	4	18	37	35	24
vB_EamM_Asesino	137	2	3	0	6	3	22	37	33	23
vB_EamM_Stratton	128	3	4	2	7	4	21	38	33	23
phiEaH2	131	3	4	2	6	4	21	37	32	22
PhiEaH1	128	3	1	1	7	4	15	30	29	28
vB_Ea277G	127	3	1	1	7	4	15	30	29	28
vB_EamM_Machina	125	4	5	0	8	4	23	36	33	24
vB_EamM_Huxley	126	4	5	0	7	4	23	35	32	25
pEa_SNUABM_8	127	3	5	0	7	4	20	43	41	26
pEa_SNUABM_4	127	3	5	0	7	4	20	43	41	26
pEa_SNUABM_9	122	3	5	1	8	4	21	41	39	25
vB_EamM_Chris	117	3	5	1	8	4	23	40	37	26
pEa_SNUABM_43	121	4	5	1	8	4	23	39	37	23
pEa_SNUABM_42	121	4	5	1	8	4	23	39	37	23
vB_EamM_Caitlin	126	3	5	1	8	4	22	36	33	26
vB_EamM_Parshik	126	4	5	0	7	4	23	35	32	25
pEa_SNUABM_6	126	4	5	0	8	5	22	37	35	23
pEa_SNUABM_10	132	4	5	0	8	4	21	36	34	23
vB_EamM_Joad	126	2	3	2	2	5	18	30	32	20
vB_EamM_RisingSun	129	2	3	1	2	5	17	30	32	21
pEa_SNUABM_29	121	3	5	1	9	4	22	47	43	25
pEa_SNUABM_11	143	3	7	2	6	5	20	40	36	22
vB_EamM_Kwan	146	2	6	2	6	6	22	33	30	21
Wellington	144	2	5	3	7	6	22	33	30	21
Demerecviridae										
KEY	76	0	1	2	9	7	19	9	19	38
Eneladusvirus										
pEa_SNUABM_12	234	4	11	8	8	10	28	74	63	106
pEa_SNUABM_49	228	3	10	11	8	13	27	74	63	106
pEa_SNUABM_50	234	4	11	8	8	8	27	74	63	103
pEa_SNUABM_44	232	4	11	7	8	7	27	74	63	108
pEa_SNUABM_47	231	4	11	8	8	7	27	74	63	105
pEa_SNUABM_19	231	4	11	8	8	7	27	74	63	106
Inoviridae										
PEar6	0	2	0	1	0	0	2	5	1	0
PEar4	0	1	0	1	0	0	2	5	1	0
PEar2	0	1	0	1	0	0	2	5	1	0
PEar1	0	1	0	1	0	0	2	5	1	0
Myosmarvirus										
vB_EamM_TropicalSun	60	0	1	1	1	1	7	5	14	11
Ounavirinae										
Omen	38	0	1	5	10	1	14	10	11	26
Tian	40	0	1	4	10	1	14	10	11	26
Roscha1	42	0	1	3	10	2	13	10	11	25
vB_EamM-M7	39	0	1	4	10	2	14	10	11	24
vEam_W_28	40	0	1	3	10	1	13	10	11	25
vEam_S_24	40	0	1	3	10	1	13	10	11	25
vEam_PM_21	40	0	1	3	10	1	13	10	11	25
vEam_PM_27	40	0	1	3	10	1	13	10	11	25
vEam_PM_6	40	0	1	3	10	1	13	10	11	25
vEam_W_25	40	0	1	3	10	1	13	10	11	25
Panisse	39	0	1	3	10	1	14	11	11	26
phiEa21-4	42	0	1	3	10	1	13	10	11	25
phiEa104	39	0	1	4	11	1	13	10	11	26
SunLIRen	40	0	1	3	10	1	13	10	11	26
Pistou	41	0	1	3	10	1	13	10	11	25
FIfi106	39	0	1	4	11	1	13	10	11	25
Rouille	40	0	1	3	10	1	13	10	11	25
Hena2	40	0	1	3	10	1	13	10	11	25
Pavtokvirus										
Pavtok	22	2	2	3	3	3	2	8	6	9
PEp14	27	2	2	2	2	3	3	7	6	9
Stean	22	3	3	2	3	3	3	7	6	10
Peduoviridae										
ENT90	4	2	0	4	3	2	1	8	14	0
Rivsvirus										
pEa_SNUABM_5	273	2	2	2	4	11	16	7	20	9
Sasquatchvirus										
vB_EamM_Y3	255	3	2	3	3	10	16	8	22	11
pEp_SNUABM_52	257	3	2	3	2	10	15	8	21	12
Schitoviridae										
phiEaP8	30	1	1	2	6	3	9	7	3	17
Kuerle	35	1	1	2	6	3	9	7	3	20
Ea9-2	40	2	1	2	6	3	9	7	3	19
vB_EamP_Rexella	38	2	1	2	6	4	9	7	3	18
Fifi067	35	1	1	2	6	3	9	7	3	19
vB_EamP-S6	67	0	0	0	5	2	8	8	3	13
Pastis	76	1	0	0	4	2	8	7	3	11
vB_EamP_Gutmeister	33	1	1	2	6	2	9	7	3	18
Snuvirus										
pEa_SNUABM_7	267	2	3	2	3	9	15	9	23	8
Straboviridae										
Cronus	137	4	4	9	14	11	26	35	21	44
Vequintavirinae										
Hena1	92	2	4	6	7	6	21	18	21	67
pEa_SNUABM_56	92	3	4	6	7	7	18	16	17	76
pEp_SNUABM_01	91	3	4	5	7	7	18	16	18	76
pEa_SNUABM_55	88	3	4	6	7	7	20	16	17	77
Yoloswagvirus										
vB_EamM_Yoloswag	248	3	3	3	6	11	16	9	23	7
Unknown										
pEa_SNUABM_48	208	2	0	1	5	4	22	36	39	32
pEa_SNUABM_37	211	3	0	1	6	4	23	33	36	32
pEa_SNUABM_54	188	2	2	1	3	5	19	30	31	35
pEa_SNUABM_21	264	3	3	3	3	9	15	8	23	9
pEa_SNUABM_13	262	2	3	3	3	8	15	9	24	10
pEa_SNUABM_34	264	3	3	2	3	9	15	8	22	10
pEa_SNUABM_14	264	2	3	3	3	9	15	9	23	9
pEa_SNUABM_45	264	3	3	3	3	9	15	8	22	10
pEa_SNUABM_46	264	3	3	3	3	9	15	8	22	10
pEa_SNUABM_25	262	2	3	3	3	9	15	9	23	9
phiEt88	39	0	1	1	5	4	4	5	9	12

Note: Phage Scope analysis of phage protein functions; sequence data retrieved from NCBI.

**Table 3:** Genomic characterization of *E. amylovora* phages from the currently available literatures.

Phage	Complete Genome Acc No.	Size (bp)	GC Content (%)	ORFs (tRNA)
pEp_SNUABM_01	MN184887	147,321	48.7	249 (26)
pEa_SNUABM_55	OP480062	146,979	48.8	247 (25)
pEp_SNUABM_03	MT822284.1	39,879	52.1	52
pEp_SNUABM_04	MT822285.1	39,649	52.2	52
pEp_SNUABM_11	MT822287.1	39,626	52.1	49
pEp_SNUABM_12	MT822288.1	39,980	51.2	50
pEp_SNUABM_08	MN184886	62,715	57.2	79 (0)
pEa_SNUABM_12	MT939486	358,115	34.4	546 (32)
pEa_SNUABM_47	MT939487	355,376	34.5	540 (35)
pEa_SNUABM_50	MT939488	356,948	34.4	540 (34)
PEar1	MT901797	6646	41.7	10
PEar2	MT901798	6651	41.7	10
PEar4	MT901799	6801	41.7	10
PEar6	MT901800	6608	41.7	11
Kuerle	OQ181210.1	75,599	48.0	85
RH-42-1	PP099880	14,942	48.2	28 (0)
Fifi106	OR284297.1	84,405	43.4	114 (26)
vB_EamM_RAY	KU886224	271,182	49.9	317
vB_EamM_Special G	KU886222	273,224	49.8	321
key	MZ616364	115,651	39.0	182 (27)
Omen	PP278848	85,304	43.7	133
vB_EamM_Simmy50	NC_041974.1	271,088	49.9	322
vB_EamM_Y3	KY984068	261,365	47.2	333 (0)
phiEaP-8	MH160392	75,929	46.8	785 (5)
vB_EamP_Pavtok	MH426726	61,401	62.0	62 (0)
vB_EamM_SunLIRen	MH426725	84,559	43.8	141 (22)
vB_EamM_Wellington	MH426724	244,950	52.1	295 (8)
vB_EamM_Asesino	KX397364	246,290	51.2	289 (12)
vB_EamM_Alexandra	MH248138	266,532	50.1	349 (0)
vB_EamM_Bosolaphorus	MG655267	272,228	49.8	321 (1)
vB_EamM_Desertfox	MG655268	272,458	49.9	320 (0)
vB_EamM_Mortimer	MG655270	273,914	49.8	325 (1)
vB_EamM_MadMel	MG655269	275,000	49.7	321 (0)
vB_EamP-S2	NC_047917.1	45,495	49.8	49 (0)
vB_EamM-Bue1	NC_048702.1	164,037	50.2	175 (1)
PhiEa2809	KP037007	162,160	50.3	145 (1)
PhiEaH1	KF623294	218,339	52.3	241
PhiEaH2	JX316028	243,050	51.3	262
phiEa1H	FQ482084	45,522	49.7	50 (0)
phiEa100	FQ482086	45,554	49.7	50 (0)
phiEa104	FQ482083	84,564	43.8	118 (24)
vB_EamP_S6	HQ728266	74,669	52.1	115
vB_EamP_Y2	HQ728264	56,621	44.2	90
vB_EamP_M7	HQ728263	84,694	43.4	117
vB_EamP_L1	HQ728265	39,282	51.9	49
vB_EamP_Cutmeister	KX098391	71,173	46.9	84 (8)
vB_EamP_Frozen	KX098389	75,147	46.9	92 (8)
vB_EamP_Rexella	KX098390	75,448	46.9	92 (7)
vB_EamM_Deimos-Minion	KU886225	273,501	49.9	326 (0)
vB_EamM_Caitlin	KX397365	241,147	52.2	271 (7)
vB_EamM_ChrisDB	KX397366	244,840	49.4	277 (11)
vB_EamM_EarlPhilliplv	KX397367	223,935	50.6	241 (0)
vB_EamM_Huxley	KX397368	240,761	51.1	271 (9)
vB_EamM_Kwan	KX397369	246,390	52.1	285 (8)
vB_EamM_Machina	KX397370	241,654	51.0	272 (9)
vB_EamM_Parshik	KX397371	241,050	51.0	271 (10)
vB_EamM_Phobos	KX397372	229,501	49.1	247 (0)
vB_EamM_Stratton	KX397373	243,953	51.3	276 (12)
vB_EamM_Yoloswag	KY448244	259,700	46.9	334 (0)
vB_EamM_RisingSun	MF459646	235,108	48.32	243 (0)
vB_EamM_Joad	MF459647	235,374	48.29	245 (0)
phiEa21-4	EU710883.1	84,576	43.8	117 (26)
Ea35-70	KF806589	271,084	49.9	318 (1)

Note: Data obtained from NCBI (National Center for Biotechnology Information) databases.

**Table 4:** The lytic activity of some *E. amylovora* phages.

Phages	Lytic/Lysogenic	Latent Period (min)	Burst Size (PFU/Host Cell)	References
pEp_SNUABM_01	Lytic	20	67	[47]
pEp_SNUABM_55	Lytic	20	65	[47]
pEp_SNUABM_03	Lytic	10	76	[50]
pEp_SNUABM_08	Lytic	40	20	[31]
pEa_SNUABM_12	Lytic	40	17.51 ± 1.48	[45]
pEa_SNUABM_47	Lytic	40	19.94 ± 3.31	[45]
pEa_SNUABM_50	Lytic	40	15.51 ± 1.46	[45]
PEar1	Lysogenic	10	280	[10]
PEar2	Lysogenic	10	280	[10]
PEar4	Lysogenic	10	280	[10]
PEar6	Lysogenic	10	280	[10]
Kuerle	Lytic	50	240	[43]
RH-42-1	Lytic	10	207	[9]
vB_EamM_Deimos-Minion	Lytic	180–240	4.6–4.9	[24]
Fifi106	Lytic	20	310 ± 30	[48]
Fifi044	Lytic	40	20	[71]

## Data Availability

All data supporting the conclusions of this article are included in this article.
